# Arenobufagin Induces Apoptotic Cell Death in Human Non-Small-Cell Lung Cancer Cells via the Noxa-Related Pathway

**DOI:** 10.3390/molecules22091525

**Published:** 2017-09-11

**Authors:** Liang Ma, Yindi Zhu, Sheng Fang, Hongyan Long, Xiang Liu, Zi Liu

**Affiliations:** Department of Chemical Biology and Pharmaceutical Engineering, School of Chemistry and Chemical Engineering, Anhui University of Technology, Ma’anshan 243002, Anhui, China; alexingma@163.com (L.M.); zhuyindi0805@163.com (Y.Z.); fngseng12345@163.com (S.F.); longhongyan1995@163.com (H.L.)

**Keywords:** non-small-cell lung cancer, arenobufagin, Noxa, Mcl-1, p53

## Abstract

Arenobufagin, an active component isolated from the traditional Chinese medicine Chan Su, exhibits anticancer influences in several human malignancies. However, the effects and action mechanisms of arenobufagin on non-small-cell lung cancer (NSCLC) are still unknown. In this study, we reported that arenobufagin acted through activation of Noxa-related pathways and promoted apoptotic cell death in human NSCLC cells. Our results revealed that arenobufagin-induced apoptosis was caspase-dependent, as evidenced by the fact that caspase-9, caspase-3 and poly (ADP-ribose) polymerase (PARP) were cleaved, and pretreatment with a pan-caspase inhibitor Z-VAD-FMK inhibited the pro-apoptosis effect of arenobufagin. Mechanistically, we further found that arenobufagin rapidly upregulated the expression of the pro-apoptosis protein Noxa, and abrogated the anti-apoptosis protein Mcl-1, a major binding partner of Noxa in the cell. More importantly, the knockdown of Noxa greatly blocked arenobufagin-induced cell death, highlighting the contribution of this protein in the anti-NSCLC effects of arenobufagin. Interestingly, arenobufagin also increased the expression of p53, a direct transcriptional activator for the upregulation of the Noxa protein. Taken together, our results suggest that arenobufagin is a potential anti-NSCLC agent that triggers apoptotic cell death in NSCLC cells through interfering with the Noxa-related pathway.

## 1. Introduction

Lung cancer continues to be the most common cause of cancer death in the world, responsible for nearly one cancer death in five [1]. Non-small-cell lung cancer (NSCLC), which primarily includes adenocarcinoma and squamous cell carcinoma, accounts for approximately 85% of all cases of lung cancer. Despite advances in early detection, NSCLC is often diagnosed at an advanced stage, or a locally advanced stage. Chemotherapeutic agents have provided the standard regimen backbone for these patients, as well as combination chemotherapy. However, as a result of resistance, the prognosis for NSCLC patients is still poor, with a 5-year survival rate of less than 15% [2,3]. Therefore, there is an urgent need to develop more effective therapies for NSCLC patients.

Apoptosis pathways are typically dysregulated in many human cancers and can be therapeutically exploited for cancer treatment. In the past decade, the discovery and development of novel small-molecule inhibitors targeting apoptosis have been widely reported. There are two major apoptotic mechanisms: the intrinsic (mitochondrial) and the extrinsic (death receptor), which are tightly regulated by the balance between pro- and anti-apoptotic proteins [4]. The Bcl-2 protein family, including anti-apoptotic proteins (i.e., Bcl-2, Bcl-xl, BCL-w, Bcl-b, Mcl-1, and A1/Bfl-1) and two groups of pro-apoptotic proteins: multi-domain proteins (e.g., Bak, Bax) and BH3-only proteins (i.e., Bim, Bid, Bad, Puma, Bmf, Bik, Hrk, and Noxa), are crucial in regulating mitochondrial apoptosis [5]. Noxa was initially identified as a primary p53-responsive gene [6]. Recently, accumulating evidence indicates that Noxa is involved in the regulation of the cytotoxic effect triggered by a plethora of anticancer treatments [7,8,9]. Independent of its inherent pro-apoptotic activity, it was reported that Noxa played a critical role in regulating Mcl-1, an anti-apoptotic member of the Bcl-2 proteins family that is overexpressed in many tumor types [10,11,12,13]. Thus, activating Noxa-related pathways may represent a new treatment strategy for cancer.

Anti-cancer agents from natural products used in traditional Chinese medicines have attracted significant attention internationally [14]. Arenobufagin, one of the active ingredients of toad venom (also called Chan Su), is a traditional Chinese medicine obtained from the skin and parotid venom glands of the toad [15,16]. Arenobufagin was initially identified as a potent Na^+^/K^+^ pump inhibitor, and has the ability to block Na^+^-K^+^ pumps in cardiac myocytes [17,18]. In recent years, researchers found that arenobufagin possessed the potential for anti-tumor activity in some malignances. For instance, Zhang et al. reported that arenobufagin induced apoptosis and autophagy in human hepatocellular carcinoma (HCC) cells, via inhibition of the PI3K/Akt/mTOR pathway [19]. Deng et al. found that arenobufagin could intercalate with DNA to induce G2 cell cycle arrest through the ATM/ATR pathway in HCC cells [20]. Except in HCC, arenobufagin has also been shown to inhibit growth and induce apoptosis in human esophageal squamous cell carcinoma cells and cervical carcinoma cells [21,22]. However, the effects and mechanisms of arenobufagin on lung cancer are still not clear.

Here, we systematically evaluated the anti-cancer effect of arenobufagin on NSCLC cells. Our data demonstrated that arenobufagin significantly inhibited growth and induced apoptosis of NSCLC cells. More importantly, we reported a novel finding that activating Noxa-related pathways was critical in arenobufagin-triggered cell death. These results suggested that arenobufagin could be a promising agent for patients with NSCLC.

## 2. Results

### 2.1. Effects of Arenobufagin on NSCLC Cells

**Figure 1:**
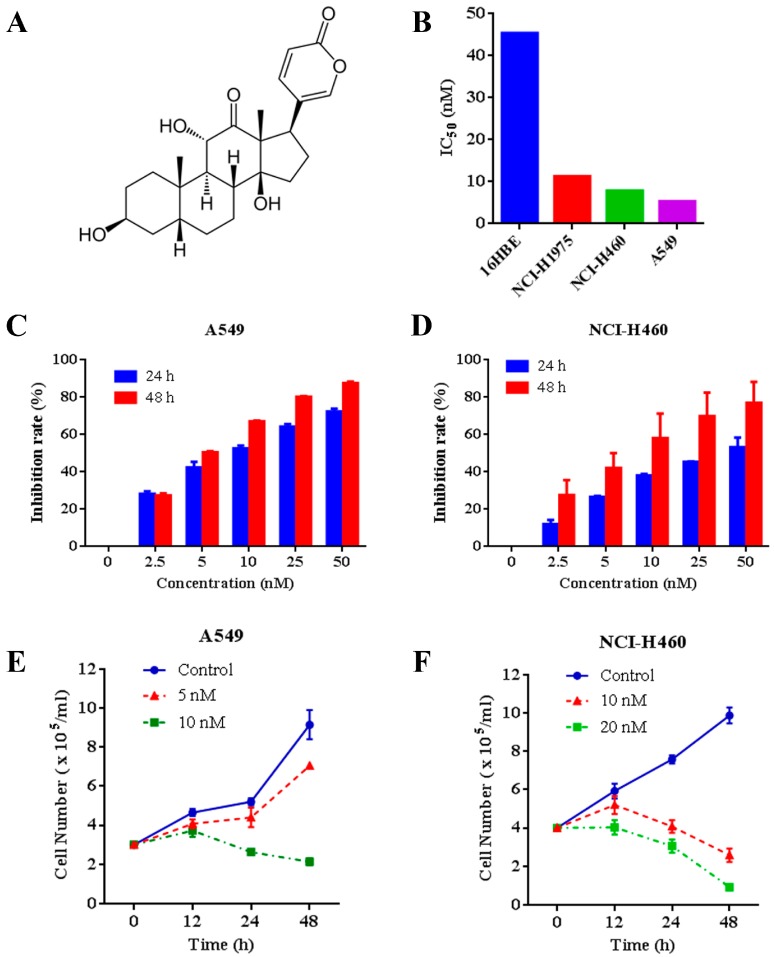
Arenobufagin decreases the cell viability in A549 and NCI-H460 cells. (**A**) The chemical structure of arenobufagin; (**B**) The IC_50_ values of arenobufagin for indicated cell lines; (**C**) The inhibitory effects of arenobufagin on A549 cells analyzed by 3-(4,5-dimethylthiazol-2-yl)-2,5-diphenyltetrazolium bromide (MTT) assay; (**D**) NCI-H460 cells were treated with various concentrations of arenobufagin for 24 and 48 h. MTT assays were conducted to measure the cell viability; (**E**) A549 cells were treated with or without arenobufagin for indicated time points, and analyzed by trypan blue exclusion assay; (**F**) Inhibitory effects of arenobufagin on cell viability of NCI-H460 cells measured by trypan blue exclusion assay.

We tested the effects of arenobufagin (Figure 1A) on multiple NSCLC cells and normal human bronchial epithelial cells. The results showed that arenobufagin exhibited higher activity to A549, NCI-H460 and NCI-H1975 NSCLC cells, with IC_50_ values around 10 nM. Arenobufagin displayed less sensitivity towards 16HBE normal human bronchial epithelial cells (more than 40 nM of the IC_50_ value) (Figure 1B), indicating its selectivity between cancer and normal cells. Thereafter, we systematically evaluated the effect of arenobufagin on A549 and NCI-H460 cells with lower IC_50_ values (Figure 1B). Our results showed that arenobufagin significantly inhibited the growth of A549 and NCI-H460 cells in a time- and dose-dependent manner by 3-(4,5-dimethylthiazol-2-yl)-2, 5-diphenyltetrazolium bromide (MTT) assay (Figure 1C,D). Trypan blue exclusion assay further demonstrated that arenobufagin reduced viable cells in A549 and NCI-H460 (Figure 1E,F). Similar results were also observed in NCI-H1975 cells (Supplementary, Figure S1A,B). These results indicated that arenobufagin exhibits great therapeutic potential in NSCLC treatment.

### 2.2. Arenobufagin Provokes NSCLC Cell Apoptosis

**Figure 2:**
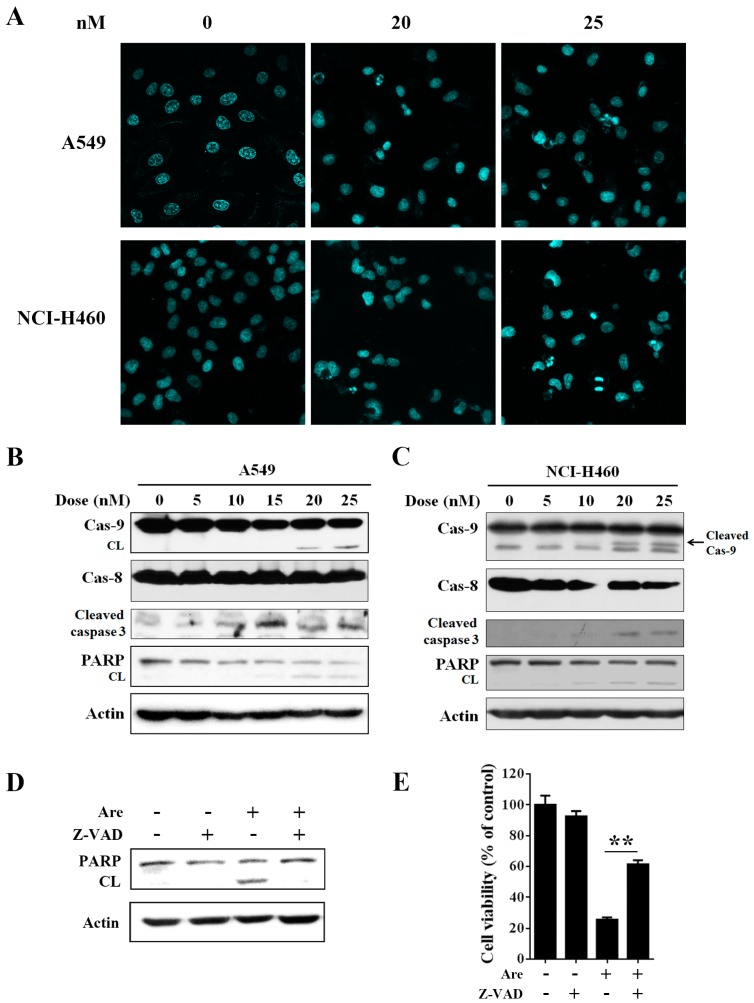
Arenobufagin induces caspase-dependent apoptosis in non-small-cell lung cancer (NSCLC) cells. (**A**) A549 and NCI-H460 cells were treated with the indicated concentrations of arenobufagin for 24 h, and apoptotic morphological changes were evaluated by Hoechst 33258 staining; (**B**,**C**) A549 and NCI-H460 cells were treated with arenobufagin at the indicated concentrations for 24 h, and protein extracts were subjected to Western blot assay with indicated antibodies; (**D**,**E**) A549 cells were pre-treated with the caspase inhibitor Z-VAD (40 μΜ) for 1 h, followed by incubation with arenobufagin (Are, 25 nM) for 24 h; (**D**) poly (ADP-ribose) polymerase (PARP) cleavage was analyzed by Western blotting; (**E**) The cell viability was detected via trypan blue exclusion assay. ** *p* < 0.01, arenobufagin-treated group versus arenobufagin and Z-VAD combination group.

We next examined the potential effect of arenobufagin on NSCLC cell apoptosis. A549 and NCI-H460 cells were treated with the indicated doses of arenobufagin for 24 h. Hoechst 33258 staining showed that arenobufagin-induced apoptotic chromatin condensation and DNA fragmentation were clearly observed (Figure 2A). We next detected the alteration of apoptosis-related proteins. As shown in Figure 2B,C, arenobufagin treatment induced a typical processing of caspase-9, caspase-3 and a classical pattern of poly (ADP-ribose) polymerase (PARP) cleavage, while it had little effect on caspase-8 cleavage in A549 and NCI-H460 cells. We also observed caspase-9 and PARP cleavage in NCI-H1975 cells (Supplementary, Figure S1C). Notably, A549 cell apoptosis, triggered by arenobufagin, was greatly inhibited after the pan-caspase inhibitor Z-VAD-FMK treatment, as revealed by Western blot assay and trypan blue exclusion assay (Figure 2D,E). These results indicated that arenobufagin induces caspase-dependent apoptotic death in NSCLC cells.

### 2.3. Arenobufagin Regulates Noxa and Mcl-1 in NSCLC Cells

**Figure 3:**
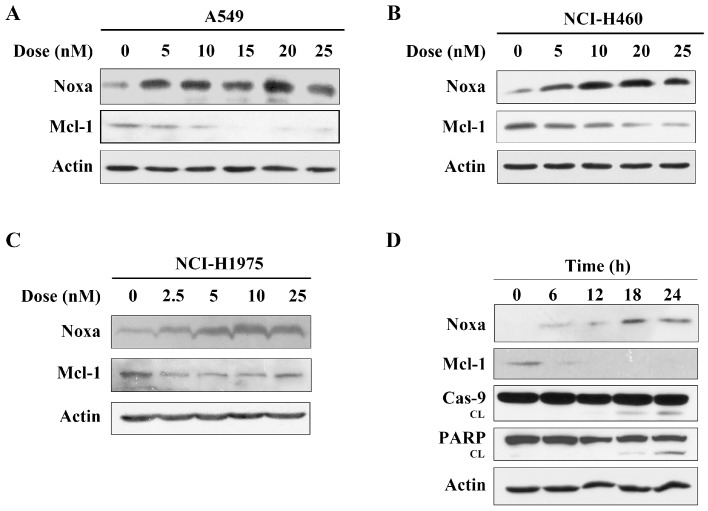
Arenobufagin up-regulates Noxa and down-regulates Mcl-1 in NSCLC cells. (**A**,**B**) A549 and NCI-H460 cells were incubated with the indicated concentrations of arenobufagin for 24 h, and the protein expression levels of Noxa, Mcl-1, and Actin were determined by Western blotting. The level of actin was used as a loading control; (**C**) Noxa up-regulation and Mcl-1 down-regulation were determined using a Western blot analysis in NCI-H1975 cells; (**D**) A549 cells were treated with arenobufagin at 20 nM for the indicated time points, and the expression of Noxa, Mcl-1, caspase-9, PARP and Actin were analyzed by Western blotting. The level of actin was used as a loading control.

The data above showed that arenobufagin induced the cleavage of the caspase-9 protein, which indicated that the intrinsic (mitochondria-mediated) apoptotic pathways were activated by arenobufagin. It was reported that the Bcl-2 protein family represented the key regulatory node of mitochondrial apoptosis [5,9]. We then detected the expression of Bcl-2 family proteins. Interestingly, we found that after treatment with arenobufagin, Noxa protein, an important mediator of the mitochondrial apoptosis pathway, was significantly increased in A549 cells (Figure 3A). Early studies showed that Noxa had the most restricted potential to neutralize Mcl-1, and later evidence suggested that Noxa upregulation promoted the degradation of the Mcl-1 protein, an anti-apoptotic member of the Bcl-2 proteins family [11,13]. It was reported that modulation of Noxa and Mcl-1 was important for compound-induced anti-cancer effects [7,8,23]. We then detected a change of Mcl-1 in A549 cells, and found that arenobufagin treatment dramatically downregulated the expression of Mcl-1 (Figure 3A). Arenobufagin also increased the expression of Noxa and abrogated Mcl-1 in NCI-H460 and NCI-H1975 cells, which were consistent with the results from A549 cells (Figure 3B,C). Further studies demonstrated that the upregulation of Noxa and reduction of Mcl-1 occurred within 6 h, while caspase-9 and PARP proteins were cleaved after 12 h, indicating that the Noxa/Mcl-1 pathway was associated with arenobufagin-triggered apoptosis in NSCLC cells (Figure 3D).

### 2.4. Noxa is Required for the Inhibition Effect of Arenobufagin on NSCLC Cells

**Figure 4:**
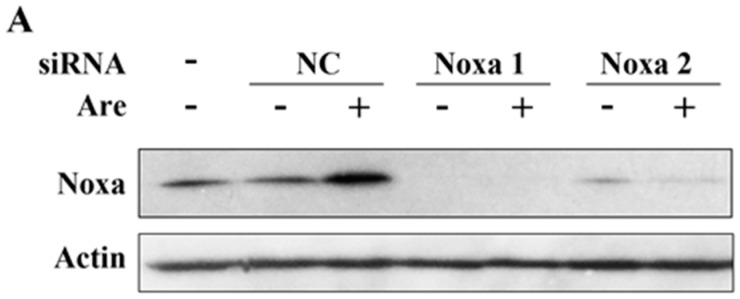

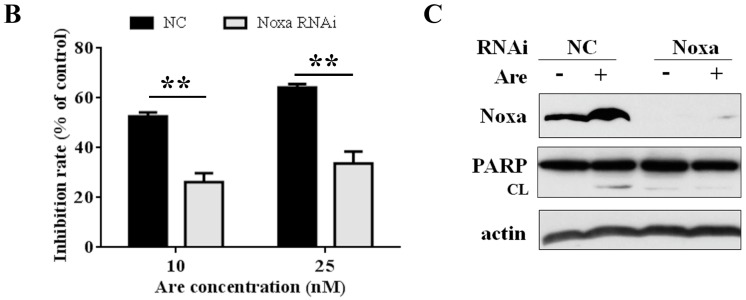
Noxa plays a critical role in the arenobufagin-induced inhibitory effect. (**A**) A549 cells transfected with control, or Noxa-specific siRNAs, for 48h were treated with or without arenobufagin (20 nM) for 24 h, lysed, and Western blot analysis was performed; (**B**,**C**) A549 cells were transfected with siRNA and treated with arenobufagin as described in (**A**); (**B**) MTT assay was conducted to analyze the cell viability of A549 cells after Noxa siRNA transfection and the following arenobufagin treatment; (**C**) Western blot assay was used to measure the expression of Noxa and PARP. ** Indicates statistical significance (*p* < 0.01).

To further confirm the importance of Noxa in the arenobufagin-induced anti-NSCLC effect, we used Noxa siRNA to downregulate its expression. As shown in Figure 4A, siRNA dramatically downregulated the expression of Noxa in A549 cells. Using MTT assay, we confirmed that Noxa played a pivotal role in the function of arenobufagin (Figure 4B). In accordance with these results, PARP cleavage triggered by arenobufagin was also inhibited after a knockdown of Noxa (Figure 4C). The results above suggested that Noxa mediates the inhibitory effect of arenobufagin on NSCLC cells and plays an essential role in this process.

### 2.5. p53 is Involved in Arenobufagin-Induced Upregulation of Noxa

**Figure 5:**
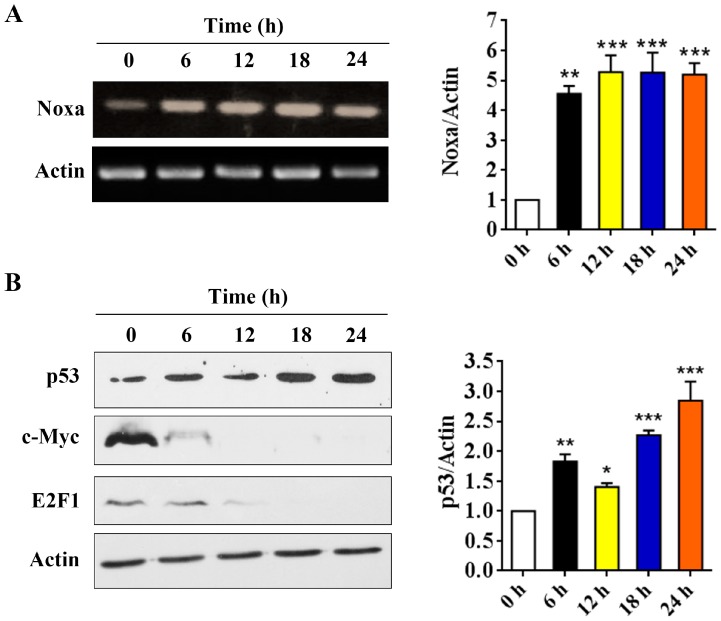

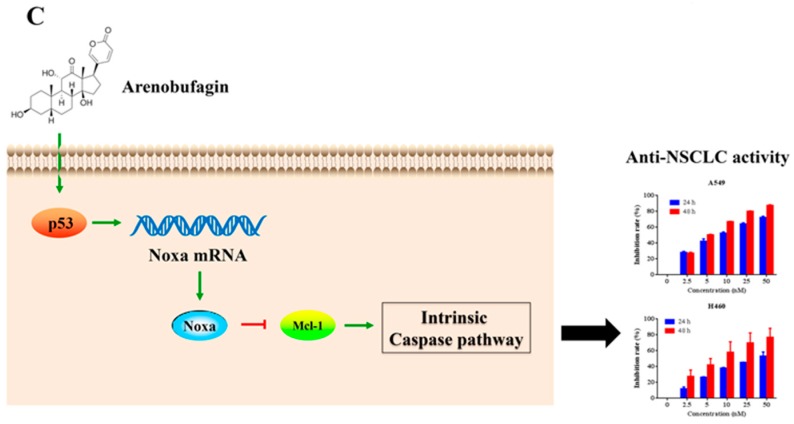
p53’s role in arenobufagin-induced Noxa upregulation. (**A**) Reverse-Transcription PCR (RT-PCR) analysis of the expression of Noxa at the mRNA level in NCI-H460 cells treated with arenobufagin (25 nM) for indicated time points; (**B**) Western blot analysis of the protein expression of p53, c-Myc, and E2F1 in NCI-H460 cells treated with arenobufagin (25 nM) for indicated time points; (**C**) The schematic model of the molecular mechanisms of anti-NSCLC activity of arenobufagin. * *p* < 0.05 compared with the control group; ** *p* < 0.01 compared with the control group; *** *p* < 0.001 compared with the control group.

Although we have shown that Noxa is upregulated after arenobufagin treatment in NSCLC cells, the mechanisms underlying this process are unclear. By using RT-PCR assay, we found that arenobufagin could upregulate Noxa at transcriptional levels in NSCLC cells (Figure 5A). Studies have shown that the transcription factors of p53, c-Myc and E2F1 can transcriptionally increase Noxa expression [6,24,25]. We then detected the expression of these proteins in NSCLC cells upon arenobufagin incubation. The results showed that p53 was significantly upregulated in NCI-H460 cells treated with arenobufagin (Figure 5B), while c-Myc and E2F1 were dramatically downregulated. This indicated that c-Myc and E2F1 were not the main mediators for the increase of Noxa that was triggered by arenobufagin. Thus, our results indicated that p53 may serve as a transcription factor for arenobufagin-induced Noxa upregulation. The possible mechanisms of arenobufagin-induced apoptosis in NSCLC cells can be summarized, as in Figure 5C.

## 3. Discussion

Toad venom (also called Chan Su), initially recorded in traditional Chinese medicine more than 1000 years ago, is the dried secretion derived from either Bufo Bufo gargarizans Cantor, or Bufo melanostictus Suhneider. Toad venom has been widely used in China as a local anesthetic, cardiotonic, antimicrobial, and antineoplastic agent for many years [26,27]. Arenobufagin, as one of the main biologically active compounds extracted from toad venom, has been showed to exhibit potential anti-cancer effects in human hepatocellular carcinoma (HCC), esophageal squamous cell carcinoma and cervical carcinoma cells [19,21,22]. However, the anti-NSCLC effects of arenobufagin are largely unknown. In this study, we demonstrated for the first time that arenobufagin is able to inhibit growth and induce apoptosis in NSCLC cells, by modulation of the Noxa-related pathway, paving the way for this compound to be exploited as a therapeutic agent in the adjunct therapy of NSCLC.

Since the 1970s, Chinese clinical trials have repeatedly shown that Huachansu, an injectable form of toad venom in physiological saline solution, has anticancer activity with low toxicity and mild adverse effects. Using a phase I clinical trial design, Meng et al. reported that no dose-limiting toxicities (DLT) were observed with the use of Huachansu at doses up to eight times higher than typically used in China [26]. As for the selective activity of a single compound from the toad venom, Lv et al. reported that arenobufagin showed lower toxicity towards human normal esophageal squamous cells, compared with esophageal squamous cell carcinoma (ESCC) cells [22]. Takai et al. also showed that bufalin, another active component of toad venom, had inhibition effects on human endometrial and ovarian cancer cells, with little toxic effect on normal cells at low doses [28]. In accordance with these studies, we found that arenobufagin significantly inhibited the growth of NSCLC cells, while displaying lower toxicity towards 16HBE normal human bronchial epithelial cells. This indicated a preference of arenobufagin in inhibiting NSCLC cells over normal human bronchial epithelial cells.

Researchers showed that arenobufagin induced apoptosis in hepatocellular carcinoma, esophageal squamous cell carcinoma and cervical carcinoma cells [19,21,22]. Our data demonstrated that arenobufagin also induced apoptosis in NSCLC cells. It was reported that the mechanisms of arenobufagin-induced apoptosis were implicated in the PI3K/Akt/mTOR pathways and the p53 pathway [19,22]. In this study, we found a novel mechanism that arenobufagin could induce mitochondria-mediated apoptosis in NSCLC cells via regulation of Noxa-related pathways. Noxa and Mcl-1 are members of Bcl-2 protein family. Noxa is well known for its pro-apoptotic effect, while Mcl-1 is a classic anti-apoptotic protein. They have opposing apoptotic activities that mediate cell death. The importance of Noxa and Mcl-1 as drug targets in cancer therapy is becoming increasingly evident. Our results indicated that upregulation of Noxa and decrease of Mcl-1 were responsible for the pro-apoptosis effect of arenobufagin. In accordance with our findings, research has shown that the modulation of Noxa and Mcl-1 is critical for the cytotoxic effect of many anticancer treatments. Therefore, our study implied that targeting the Noxa/Mcl-1 pathway could serve as a new treatment strategy for NSCLC therapy.

Noxa was initially identified as a primary p53-responsive gene, and could be regulated transcriptionally in response to genotoxic stress [6]. Recently, Lv et al. reported that arenobufagin moderately increased the expression of the p53 protein and significantly enhanced its phosphorylation in ESCC cells [22]. Interestingly, we found that arenobufagin also increased p53, and that the activation of p53 might be involved in arenobufagin-induced upregulation of Noxa in NSCLC cells. Noxa appears to be crucial for fine-tuning cell death decisions by targeting the Mcl-1 protein for degradation. This event appears to be critical for cell death induction along the mitochondrial Bcl2-regulated apoptosis pathway, in response to factor deprivation or DNA damage [10,29,30]. P53 was widely reported to be potentially activated by DNA damage [31,32]. A recent study also showed that arenobufagin intercalated with DNA and induced DNA damage, as well as a transient increase in transcriptionally active p53 in HCC cells [20]. Therefore, our results suggested that arenobufagin might regulate the p53/Noxa/Mcl-1 pathway (Figure 5C).

## 4. Materials and Methods

### 4.1. Cell Lines and Cell Culture

The lung cancer lines NCI-H1975, A549 and NCI-H460 were obtained from the American Tissue Culture Collection (ATCC). Human normal bronchial epithelial cell line 16HBE was purchased from the Cell Resource Center, Chinese Academy of Medical Sciences (Beijing, China) and cultured according to standard protocols. The cells were grown in a humidified incubator containing 5% CO_2_ in air at 37 °C. A549 and NCI-H460 cells were cultured with Dulbecco's Modified Eagle's Medium (DMEM, HyClone, Logan, UT, USA) while NCI-H1975 cells were cultured in Roswell Park Memorial Institute (RPMI)-1640 (HyClone, Logan, UT, USA). Both media were supplemented with 10% fetal bovine serum (FBS, HyClone, Logan, UT, USA), 100 U/mL penicillin and 100 mg/mL streptomycin.

### 4.2. Reagents and Antibodies

Arenobufagin was purchased from MedChem Express (MedChem Express, Shanghai, China) and dissolved in dimethylsulfoxide (DMSO, Vetec) to make a stock solution at 50 mM and stored at −20 °C until used. The caspase inhibitor Z-VAD-fmk and antibodies including anti-cleaved caspase-3 and caspase-8 were obtained from the Beyotime Institute of Biotechnology (Haimen, China). The small interfering RNAs (siRNAs) were synthesized by Shanghai GenePharma Co. Ltd (Shanghai, China). Lipofectamine 2000 reagent was purchased from Invitrogen. The anti-PARP and caspase-9 antibodies were obtained from Cell Signaling Technology (Danvers, MA, USA). The antibodies including anti-Noxa, Mcl-1, p53 and c-Myc, and the secondary antibodies, including horseradish peroxidases (HRP)-conjugated goat anti-mouse and goat anti-rabbit immunoglobulin G were purchased from Santa Cruz (Dallas, TX, USA). Human E2F1 and actin antibodies were purchased from Sigma-Aldrich.

### 4.3. Cell Viability Assay

Cell viability was measured by the MTT cytotoxicity assay [33]. A549 and NCI-H460 cells were seeded into 96-well plates at a density of 5000–10,000 cells per well, with three replicate wells per group. When the cells of each well reached 70–80% confluent, the indicated concentrations of arenobufagin were added. After treatment for 24 and 48 h, the cells were incubated with 10 μL of MTT (5 mg/mL) for 2–4 h at 37 °C. Then, the medium was discarded and 150 μL of DMSO was added to each well to dissolve formazan for measurement. The optical density (OD) was measured at an absorbance wavelength of 490 nm, using a microplate reader (Thermo Fisher, Waltham, MA, USA).

### 4.4. Hoechst 33258 Staining

Characteristic apoptotic morphological changes were assessed by Hoechst 33258 staining, using the Hoechst staining kit (Beyotime, Haimen, China). In brief, cells were exposed to arenobufagin for 24 h and then fixed with fixing buffer for 10 min at room temperature. After staining with Hoechst 33258 for 10 min, the cells were observed under a confocal laser scanning microscope (Fluoview FV10i, Olympus, Tokyo, Japan) at high magnification (600×) to determine nuclei fragmentation and chromatin condensation.

### 4.5. Western Blot Analysis

After treatments, cells were lysed on ice for 30 min in RIPA buffer containing 50 mM Tris-HCl (pH 7.4), 150 mM NaCl, 1% Triton X-100, 1% sodium deoxycholate, 0.1% SDS, 1 mM Na_3_VO_4_, 1 mM NaF, and 1 mM PMSF. Then, lysates were centrifuged at 12,000× *g* for 10 min at 4 °C to obtain the supernatant extract, followed by quantitation with the bicinchoninic acid (BCA) method. After denaturation, an equal quantity of total protein per lane was separated by sodium dodecyl sulfate–polyacrylamide gel electrophoresis (SDS-PAGE), and transferred to nitrocellulose membranes. The membranes were blocked with 5% no-fat milk in Tris-buffered saline containing Tween 20 (TBST) for 1 h at room temperature and incubated with specific primary antibodies overnight at 4 °C. Following four washes (5 min each) with TBST, the membranes were incubated with the corresponding secondary antibody for another 2 h at room temperature. After extensive washing with TBST, the signals on the membranes were detected using an enhanced chemiluminescence substrate (Cwbiotech, Beijing, China). Actin served as an endogenous loading control.

### 4.6. RNA Interference

Cells (2 × 105 cells/well) were seeded in 6-well plates, and transfected with Noxa or negative control (NC) siRNA using lipofectamine 2000 following the manufacturer’s instructions. After 48 h transfection, the cells were treated with arenobufagin for an additional 24 h, followed by the Western blot and MTT assay, as previously described [34]. The siRNA sequences of Noxa were as follows: 5′-GGUGCACGUUUCAUCAAUU-3′ (Noxa 1), 5′-GGAGAUUUGGAGACAAACU-3′ (Noxa 2) [25,35].

### 4.7. RNA Extraction and Reverse-Transcription PCR

According to the manufacturer’s protocol, the total RNA of the cells was extracted using the TRIZOL Reagent (Invitrogen, Carlsbad, CA, USA) and the phenol-chloroform extraction method. cDNA synthesis was performed using PrimeScriptTM 1st Strand cDNA Synthesis Kit (TaKaRa). Primers for Noxa detection were as follows: sense primer: 5′-AAGAAGGCGCGCAAGAAC-3′; antisense primer: 5′-CGTGCACCTCCTGAGAAAAC-3′ [25]. The reaction mix contained: 2.5 µL 10 × Ex Taq Buffer, 2 µL dNTP Mixture, 200 nM forward and reverse primers, 100 ng cDNA template, 0.25 µL TaKaRa Ex Taq and ddH_2_O up to 25 µL volume. The PCR cycling conditions consisted of the following: 98 °C for 10 s for denaturation, 55 °C for 15 s for annealing and 72 °C for 30 s for extension, for a total of 30 cycles. Products of RT-PCR were separated by 1.5% agarose gel electrophoresis and detected in a gel imaging system (UVP GelMax Imager System, Upland, CA, USA).

### 4.8. Statistical Analysis

Data were collected and analyzed using GraphPad Prism 6.0 software and expressed as the mean ± standard deviation (SD). Student’s *t*-test was used to compare data between two groups. One way analysis of variance was performed to compare data of more than two groups. A value of *p* < 0.05 was considered to be statistically significant.

## 5. Conclusions

In summary, we demonstrated that arenobufagin inhibited growth and induced apoptosis in NSCLC cells. Mechanistically, we found that the activation of Noxa-related pathways might contribute to the anti-NSCLC effects of arenobufagin. Therefore, our study demonstrates that arenobufagin exhibits potent activity against NSCLC cells through a novel mechanism, which will be beneficial for the application of this compound to the treatment of NSCLC.

## Supplementary Materials

Supplementary materials are available online.
