# Supplementary material for: Arenobufagin Induces Apoptotic Cell Death in Human Non-Small-Cell Lung Cancer Cells via the Noxa-Related Pathway
Source: Molecules. 2017 Sep 11;22(9):1525. doi: 10.3390/molecules22091525 (PMC6151516; doi:10.3390/molecules22091525)
Supplement: Supplementary file 1 [file molecules-22-01525-s001.pdf]

*Article*

# Arenobufagin Induces Apoptotic Cell Death in Human Non-Small-Cell Lung Cancer Cells via Noxa-related Pathway

**Liang Ma, Yindi Zhu, Sheng Fang, Hongyan Long, Xiang Liu * and Zi Liu ***

Department of Chemical Biology and Pharmaceutical Engineering, School of Chemistry and Chemical Engineering, Anhui University of Technology, Ma'anshan, Anhui 243002, China; alexingma@163.com (L.M.); zhuyindi0805@163.com (Y.Z.); fngseng12345@163.com (S.F.); longhongyan1995@163.com (H.L.)

* Correspondence: liuxiang@ahut.edu.cn (X.L.); tarring126@126.com (Z.L.); Tel.: +86-555-2311-551

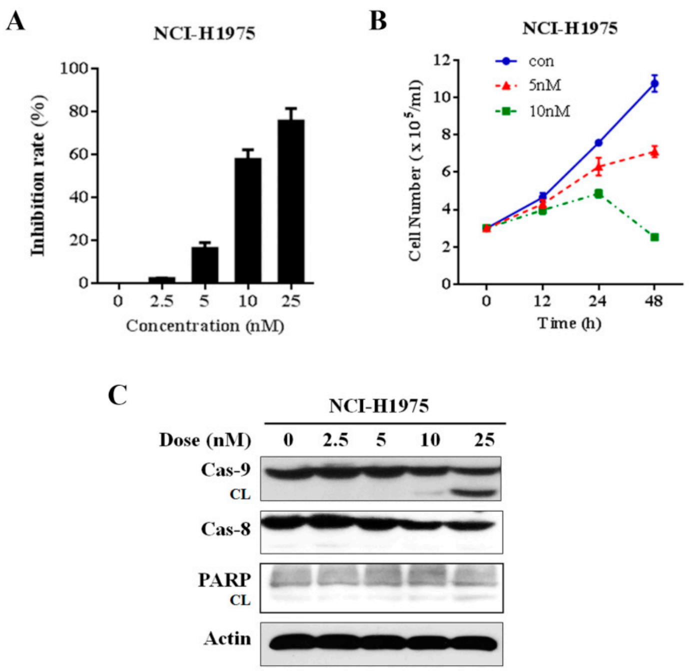


**Figure S1.** Anti-NSCLC activity of arenobufagin on NCI-H1975 cells. (**A**) The inhibitory effects of arenobufagin on NCI-H1975 cells analyzed by 3-(4, 5-dimethylthiazol-2-yl)-2, 5-diphenyltetrazolium bromide（MTT）assay; (**B**) Inhibitory effects of arenobufagin on cell viability of NCI-H1975 cells measured by trypan blue exclusion assay; (**C**) NCI-H1975 cells were treated with indicated doses of arenobufatin, and the activation of Caspse-9 (Cas-9), Caspse-8 (Cas-8), and PARP was measured by western blot analysis.
